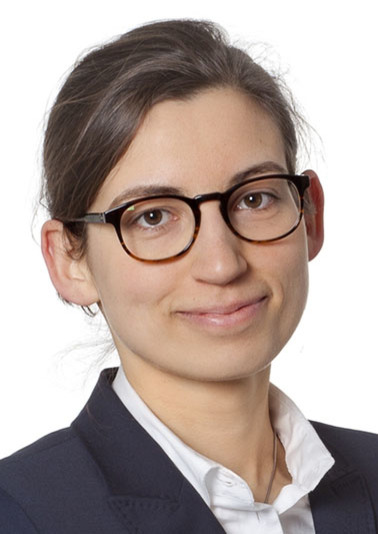# Recent Advances in Light Energy Conversion with Biomimetic Vesicle Membranes

**DOI:** 10.1002/cbic.202100220

**Published:** 2021-07-14

**Authors:** Novitasari Sinambela, Julian Bösking, Amir Abbas, Andrea Pannwitz

**Affiliations:** ^1^ Institut für Anorganische Chemie I Universität Ulm Albert-Einstein-Allee 11 89081 Ulm Germany

**Keywords:** energy transfer, liposomes, luminescence, photocatalysis, vesicles

## Abstract

Lipid bilayer membranes are ubiquitous in natural chemical conversions. They enable self‐assembly and compartmentalization of reaction partners and it becomes increasingly evident that a thorough fundamental understanding of these concepts is highly desirable for chemical reactions and solar energy conversion with artificial systems. This minireview focusses on selected case studies from recent years, most of which were inspired by either membrane‐facilitated light harvesting or respective charge transfer. The main focus is on highly biomimetic liposomes with artificial chromophores, and some cases for polymer‐membranes will be made. Furthermore, we categorized these studies into energy transfer and electron transfer, with phospholipid vesicles, and polymer membranes for light‐driven reactions.

## Graphical abstract

**Vesicles** can be used for the compartmentalization of chemical reactions and self‐assembly of reaction partners. This minireview highlights recent advances in biomimetic and light‐induced energy conversions with liposomes and polymersomes, relevant in the context of energy transfer, electron transfer, photocatalysis and artificial photosynthesis.

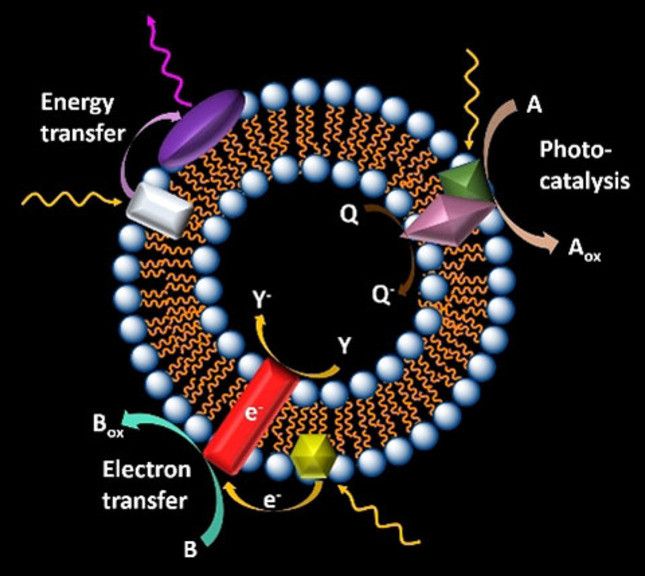

## 1 Introduction

**Figure 1:**
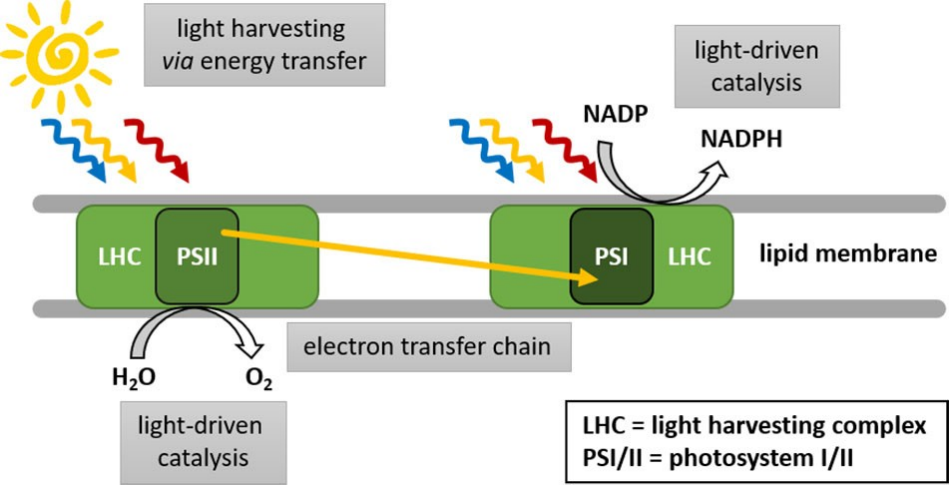
The LHCs as well as PSI and PSII play a central role in valorization of sunlight energy.

**Figure 2:**
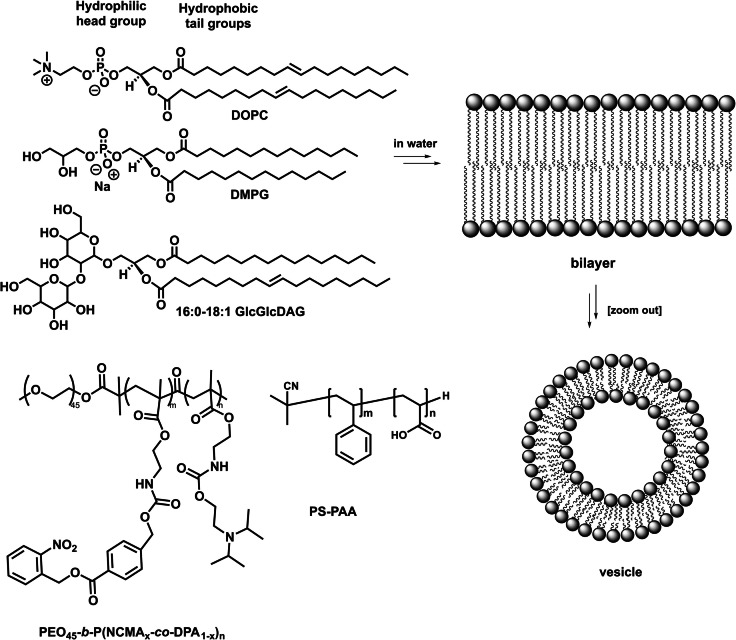
Examples for bilayer vesicle forming molecules: Phospholipids DOPC (1,2‐dioleoyl‐*sn*‐glycero‐3‐phosphocholine) and DMPG (1,2‐dimyristoyl‐*sn*‐glycero‐3‐phosphoglycerol), galactolipid 16 : 0–18 : 1 GlcGlcDAG (1‐palmitoyl‐2‐oleoyl‐3‐bis(β‐*d*‐glucosyl)‐*sn*‐glycerol), and the block copolymers PEO_45_‐*b*‐P(NCMA_x_‐*co*‐DPA_1‐x_)_n_ (PEO=polyethyleneoxide, NCMA=2‐nitrobenzyl ester‐photocaged carboxyl monomer, DPA=tertiary amine‐containing monomer with carbamiate side linkage) and PS‐PAA (polystyrene‐polyacrylic acid).

Chemical conversions associated to phospholipid bilayer membranes are ubiquitous in nature, and it becomes increasingly evident that a thorough fundamental understanding of this important class of self‐assembly is highly desirable for chemical reactions and solar energy conversion, as it enables for compartmentalization of reactions and self‐assembly of reaction partners.[^1^, ^2^, ^3^] Examples for compartmentalized chemical reactions were demonstrated using interfaces of two liquids, lipid bilayer membranes or similar soft materials.[^3^, ^4^, ^5^, ^6^, ^7^] The aspect of self‐assembly was shown to be promising for the reduction of CO_2_, proton reduction to produce H_2_, and water oxidation.[^8^, ^9^, ^10^, ^11^, ^12^, ^13^] In nature, the most relevant light‐driven reactions are taking place during photosynthesis and in the thylakoid membrane of chloroplasts. Precisely oriented chromophores in transmembrane proteins are harvesting light and using it to induce a transmembrane charge transfer for compartmentalized water oxidation as well as nicotinamide adenine dinucleotide phosphate (NADP) reduction to NADPH and adenosine triphosphate (ATP) generation.[^14^, ^15^, ^16^] The most interesting transmembrane protein complexes for sunlight energy conversion are the light harvesting complexes (LHC) as well as photosystem I and II (PSI and PSII, see Figure 1). The LHC absorb light of various energies and transfer this energy to the central chromophores in PSII and PSI. Charge separation is induced and electrons are transferred between the photosystems, to finally provide electrons for NADP reduction on one side, while water is oxidized on the other side of the membrane. Both the protein scaffold and the membrane composition have an influence on the effectiveness of these processes in nature.[^15^, ^17^] Inspired by nature, the concept of self‐assembly and compartmentalization via lipid membranes is highly promising for synthetic chemical reactions. Examples of bilayer and vesicle forming molecules and polymers are depicted in Figure 2. The composition and properties of lipid bilayers can influence energy and electron transfer dynamics within lipid bilayers, as shown for the natural light harvesting complex,^[17]^ as well as for artificial systems in this review.

This minireview focusses on selected case studies from recent years, most of which were inspired by either membrane‐facilitated light harvesting or respective charge transfer. The main focus is on highly biomimetic liposomes with artificial chromophores, and some cases for polymer‐membranes will be made. Furthermore, we categorized these studies into energy transfer and electron transfer, with phospholipid vesicles, and polymer membranes for light‐driven reactions.

For a tutorial introduction, including detailed physicochemical aspects, the reader is referred to a recently published tutorial review,^[18]^ and ion transfers,^[19]^ and somewhat older studies were reviewed elsewhere.[^20^, ^21^]

## 2 Energy Transfer in Lipid Bilayers

As described earlier, one of the key components in natural photosynthesis is the light harvesting complex which absorbs and transfer light energy. In the following, several studies of energy transfer in lipid membranes are reviewed. Most of these studies operate via Förster resonance energy transfer (FRET), while some energy transfer mechanisms rely on collision‐induced energy transfers.

**Figure 3:**
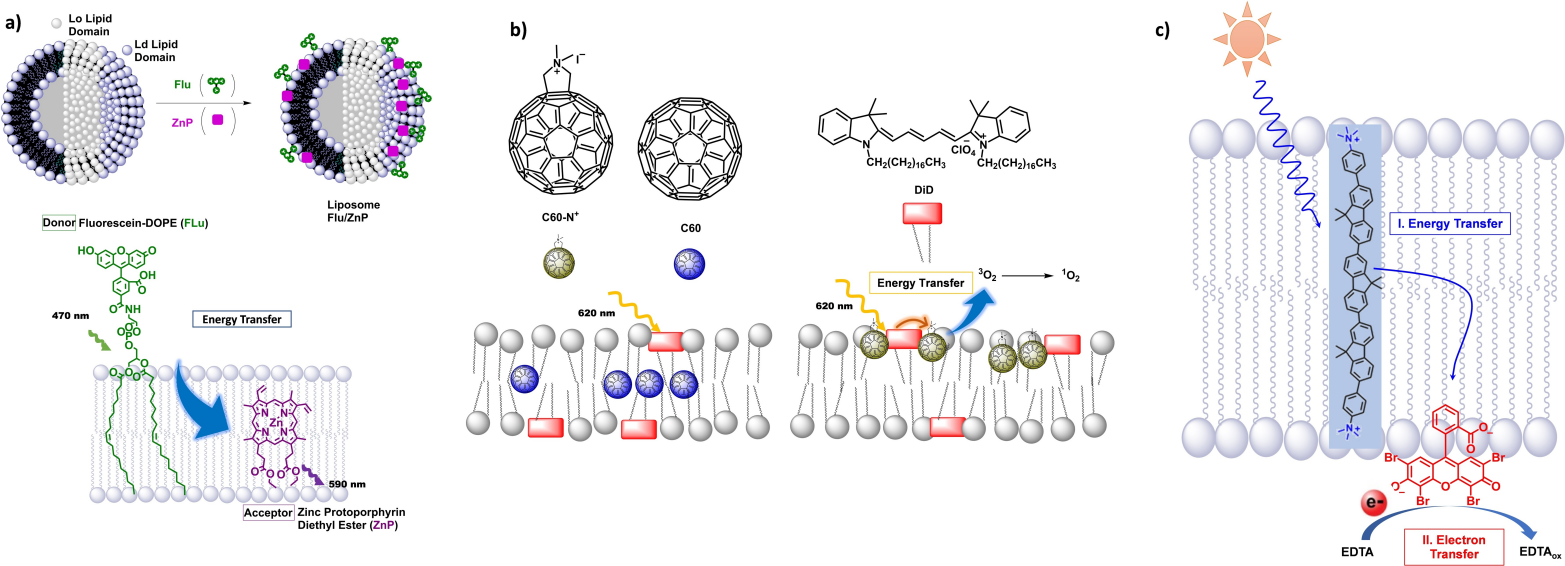
a) Accumulation of chromophores in Ld domains of liposomes for efficient energy transfer. Reproduced with permission from ref. [23]; Copyright: 2017, The Chemical Society of Japan. b) Light harvesting and energy transfer are enhanced at the membrane‐water interface using amphiphilic, strongly at 620 nm absorbing DiD as energy donor and C60‐N^+^ as energy acceptor. Reproduced with permission from ref. [24]; Copyright: 2018, Wiley‐VCH GmbH. c) Light‐absorption by a transmembrane oligoaromatic chromophore, followed energy transfer to the acceptor, eosin Y and electron transfer at the membrane interface to water. Image reproduced from ref. [25]; published 2020 by Wiley‐VCH GmbH, Creative Commons CC BY license.

Depending on the composition, lipid bilayers can form different domains. Saturated phospholipid, such as 1,2‐dipalmitoyl‐*sn*‐glycero‐3‐phosphocholine (DPPC), in combination with cholesterol form liquid ordered (Lo) domains, while unsaturated phospholipid, such as 1, 2‐dioleoyl‐*sn*‐glycero‐3‐phosphocholine (DOPC) form liquid disordered (Ld) domains.^[22]^ In order to generate a light harvesting system, efficient energy transfer, e. g. via Förster resonance energy transfer (FRET), is interesting. When constructing such a system, it must be kept in mind, that distance and distribution of donor and acceptor molecules are the main keys to achieve efficient FRET. Koshiyama and Ohba *et al*. have taken advantage of the domain specificity of selected energy donor and acceptor molecules within lipid bilayers.^[23]^ Specifically, they showed that a maximum FRET rate could be achieved with the FRET pair of a modified fluorescein and hydrophobic zinc porphyrin (see Figure 3a). Both chromophores accumulate preferably in the liquid disordered Ld domain of lipid bilayers. By adjusting the lipid composition of the liposomes, via decreasing the DOPC : DPPC ratio to 1 : 3, the Ld domains decreased in size, which lead to a higher local concentration of the chromophores within these Ld domains. No energy transfer was observed in homogeneous solution and inefficient energy transfer was observed in vesicles with only Ld domain (1 : 0 DOPC : DPPC ratio). With this concept, the authors showed an efficient way of increasing light harvesting properties within lipid bilayers using modification of domain size.^[23]^

Light harvesting via energy transfer in lipid bilayers was used by Ikeda *et al*. to increase the amount of photogenerated singlet oxygen (^1^O_2_).^[24]^ The hydrophobic oxygen sensitizer fullerene (C60) is a weak light absorber in the visible spectrum. Using lipid bilayer membranes, it was combined with the amphiphilic light harvester 1,10‐dioctadecyl‐3,3,30,30‐tetramethylindodicarbocyanine (DiD) which strongly absorbs visible light between 600 and 700 nm (Figure 3b). Due to its positive charge, the latter molecule resides at the membrane‐water interface, while the hydrophobic C60 resided in the hydrophobic region of the membrane. Adding a positive charge to the C60 via a quarternary ammonium group (C60‐N^+^, see Figure 3b) allowed for co‐assembly of both chromophores at the membrane‐water interface. Energy transfer from photoexcited DiD to hydrophobic C60 and amphiphilic C60‐N^+^ was demonstrated via luminescence quenching. It was found that the assembly of energy donor and acceptor at the membrane‐water interface strongly increased the energy transfer, from 55 % to 87 %. Likewise, the follow‐up generation of singlet oxygen strongly increased upon co‐assembling donor and acceptor at the membrane‐water interface, as shown by bleaching of the singlet oxygen probe 1,10‐anthracenediyl‐bis(methylene)dimalonic acid (ABDA) at 400 nm. The same energy transfer effect was shown for other strongly absorbing light harvester when excitation occurred at their respective absorption maximum.^[24]^ This study shows, liposomes are a powerful method to co‐assemble energy transfer couples to increase quantum yields for follow‐up reactions such as the generation of reactive oxygen species.

In another study, a biscationic oligofluorene dye with a hydrophobic core and charged headgroups was embedded into phospholipid bilayers (see Figure 3c). This dye acted as a light harvester for energy transfer. Addition of the energy acceptor eosin Y (EY^2−^) to the phospholipid bilayer membrane induced energy transfer from the oligofluorene to EY^2−^ which was observed by luminescence quenching of the donor and lighting up of the acceptor emission. The acceptor's emission indicated that EY^2−^ was in its reactive electronic excited state. In their study, the authors used this energy for a follow‐up electron transfer with the sacrificial electron donor ethylenediamine tetra acetic acid (EDTA) in solution.^[25]^

For some photochemical applications it is impossible to excite with high‐energy light such as blue or UV‐light, because light is absorbed by a thick layer of tissue or the solvent itself in a larger batch reactor. On the other hand, lower energy photons, such as green or red‐light photons, can easily cross as they are poorly absorbed by the tissue or solvent. Sometimes, involving a photosensitizer absorbing at higher wavelength (lower energy) is sufficient to perform the desired photochemistry, as described earlier in this chapter.^[24]^ However, some processes require the use of high energy photons for excitation.

**Figure 4:**
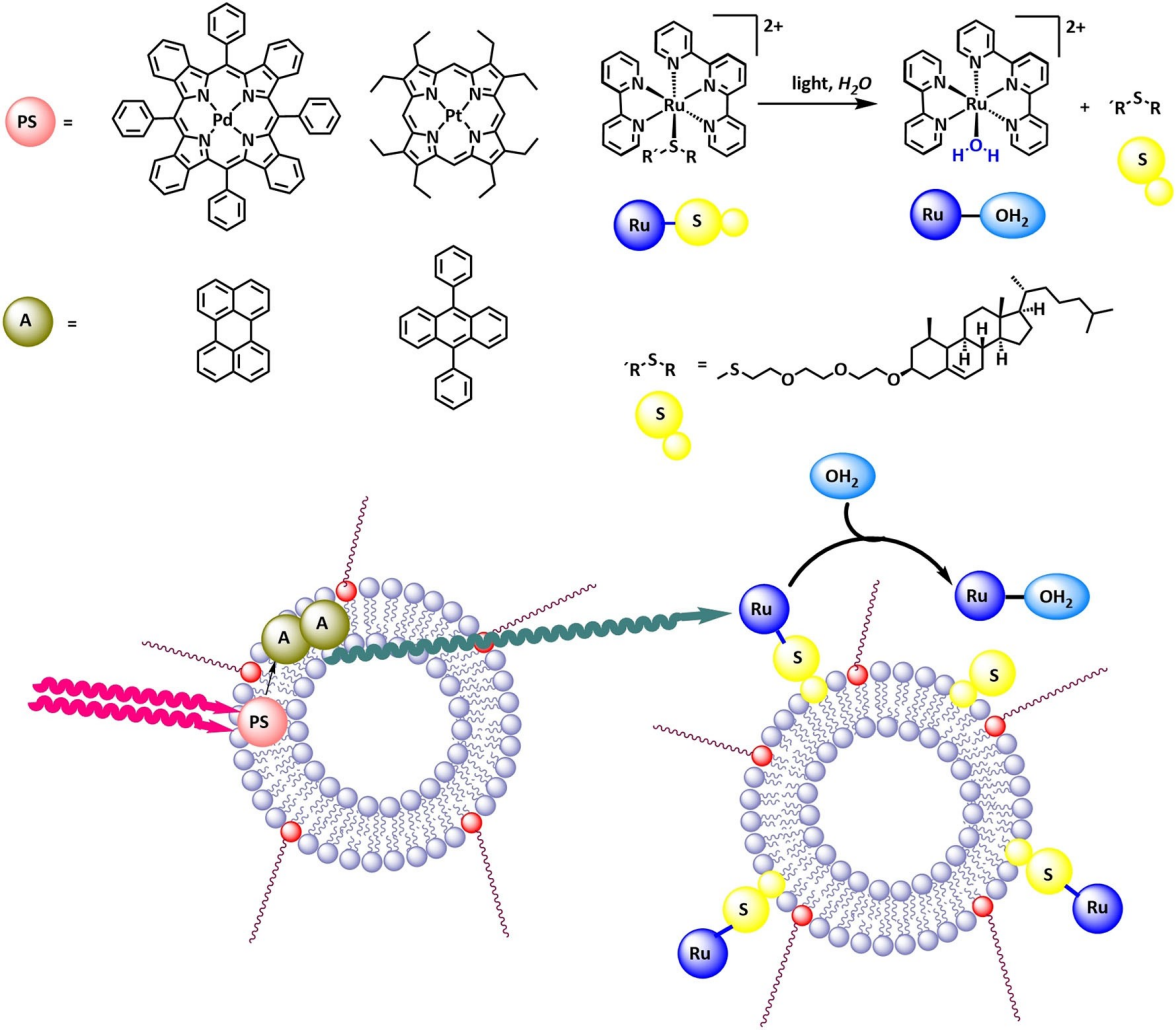
Triplet‐triplet‐annihilation‐upconversion combined with a photochemical reaction. Reproduced with permission from ref. [26]; Copyright: 2013, Wiley‐VCH GmbH. (PS=photosensitizer, A=annihilator).

Triplet‐triplet annihilation upconversion is a method to generate high energy photons from low energy photons: The photosensitizer absorbs low energy photons and undergoes intersystem crossing to generate the excited triplet state of the photosensitizer. Upon collision with an annihilator molecule, this energy is transferred to generate the triplet excited state of the annihilator molecule and the ground state of the photosensitizer. Collision of two triplet annihilator molecules leads to triplet‐triplet annihilation, whereby one molecule is promoted to the high‐energy excited singlet state, and one molecule falls back into the ground state. The singlet excited state annihilator molecule then returns to the ground state by emitting a high energy photon.^[27]^ As the annihilation process is collision based, the local concentration of the triplet excited state annihilator must be sufficiently high, which preferably happens when the triplet excited state lifetime of the annihilator molecules is long. Suitable photosensitizer‐annihilator couples are platinum or palladium porphyrins in combination with 9,10‐diphenylanthracene or perylene respectively (see Figure 4). In liposomes, these couples were shown to generate blue light from green, or red light respectively with a quantum yield of 2.3 % and 0.5 % respectively.^[26]^ It is noted, that the fluidity of the membrane played an important role in achieving high quantum yields for triplet‐triplet annihilation upconversion: By increasing the temperature above the gel‐to‐fluid phase transition temperature, the mobility within the membrane is significantly increased and blue light is generated with a high quantum yield.

Furthermore, these high energy photons were directly used in a productive ligand exchange reaction in neighboring liposomes functionalized with a cholesterol‐sulfur‐ruthenium‐complex (Figure 4). Upon photoexcitation via in situ generated blue light the sulfur‐ruthenium coordination bond is split via a photochemical ligand exchange, and the [Ru(tpy)(bpy)(OH_2_)]^2+^ (tpy=terpyridine, bpy=bipyridine) complex is released into the surrounding aqueous bulk.^[26]^ This principle could also be used with photosensitizer‐annihilator pair and photoactivatable ruthenium complexes on the same liposomes,[^28^, ^29^] and might be relevant for photochemical cancer therapy in deeper tissue,[^26^, ^27^, ^28^, ^29^, ^30^] and other photochemical conversions with liposomes.

## 3 Electron Transfer at and Across Lipid Bilayers

Light‐induced charge separation is a challenge in solar energy conversion, including artificial photosynthesis and photoredox catalysis. Oftentimes, productive charge separation is in competition with unproductive charge recombination. The following three strategies ensure the forward reaction. I) Repulsion directly after the charge transfer,^[31]^ II) rapid decomposition of one reactant by using sacrificial oxidants and reductants,^[32]^ and III) kinetic control of the inherent electron transfer rate. The latter is taken into account by Markus’ theory, which states, that there is an optimal driving force for an electron transfer as well as a reorganization barrier which play a major role in charge transfer.[^33^, ^34^, ^35^, ^36^, ^37^]

**Figure 5:**
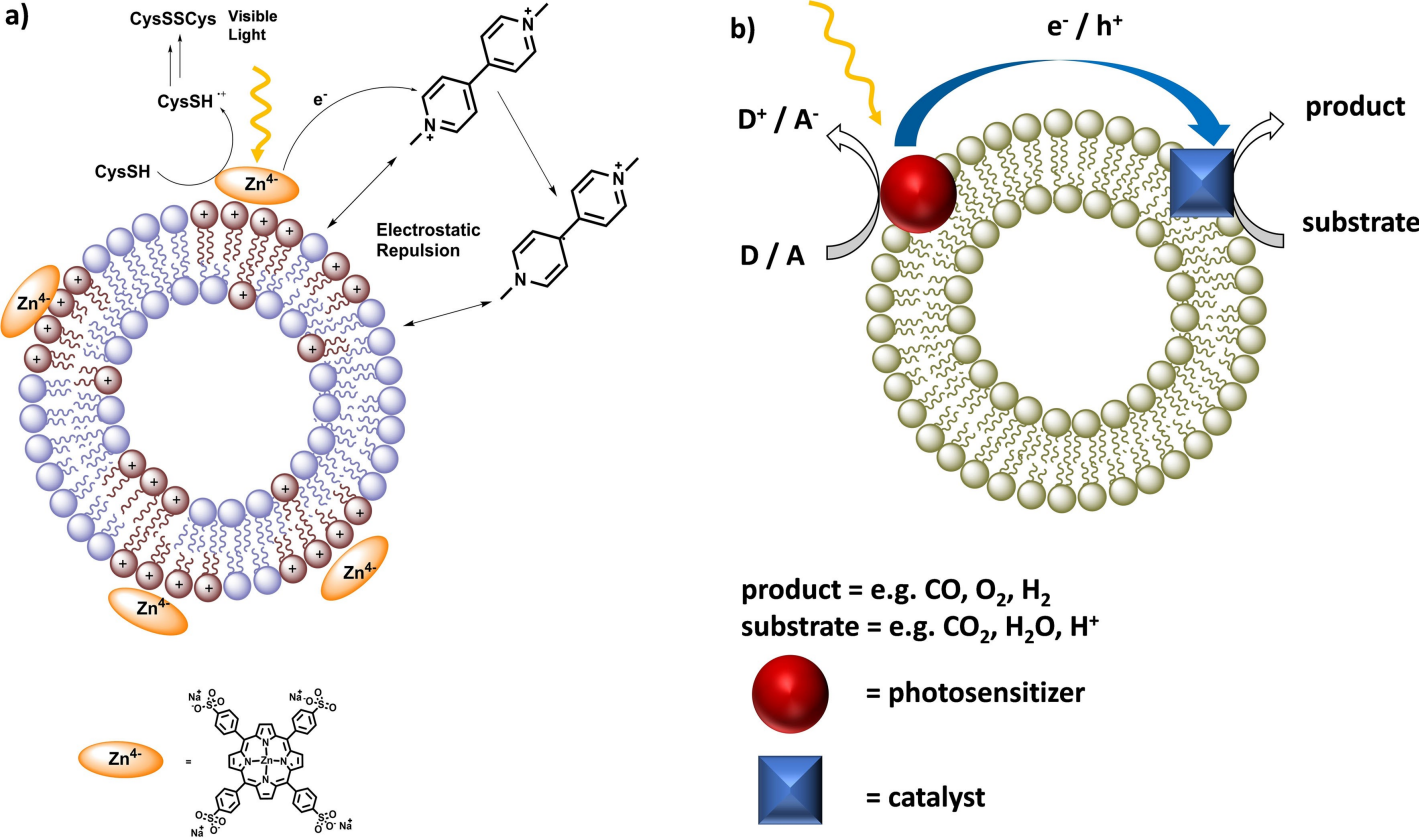
a) Light induced charge transfer from methyl viologen to photoexcited zinc porphyrin. Reproduced with permission from ref. [31]; Copyright: 2016, American Chemical Society. b) Charge transfer during photocatalysis. Upon photoexcitation the photosensitizer is oxidized or reduced by an acceptor (A) or donor (D) followed by electron (e^−^) or hole (h^+^) transfer to the active catalyst.

The repulsion strategy is especially easy to realize with liposomes. By choosing the lipid composition, the liposome surface charges can have positive, neutral or negative surface charges (for examples, see Figure 2 and Figure 5a). In one example, Bonnet *et al*. assembled positively charged liposome membrane surface with a negatively charged zinc porphyrin photosensitizer yielding an overall positively charged membrane surface.^[31]^ Irradiation of these vesicles in presence of positively charged, water soluble methyl viologen lead to bimolecular oxidative quenching. The oxidized porphyrin was re‐reduced by cysteine which acted as a sacrificial reductant. In the same experiment with all components present except for the liposomes, the forward reaction (oxidative quenching) was faster, but only little amounts of the final photoproducts were obtained, because the charge recombination within the porphyrin‐methyl viologen ion pair was too fast and prevented charge separation. However, the overall positively charged membrane surface enabled for fast repulsion of the positively charged electron acceptor radical directly after the photoinduced electron transfer, and hence effective charge separation.

Taking advantage of the self‐assembly and electron transfer dynamics between photosensitizers and catalysts at phospholipid bilayer surfaces, photocatalysis was successfully performed for water oxidation and reduction reactions, as well as CO_2_ reduction, to assemble catalysts and photosensitizers in close proximity (Figure 5b).[^10^, ^11^, ^38^, ^39^, ^40^]

Key findings of these catalysis studies that the close embedment of photosensitizer and catalyst in the membrane changes the rate limiting step of catalysis, as it increases the electron transfer rate between photosensitizer and catalyst which was shown for water oxidation reactions.^[10]^ The main reason for a fast electron transfer at liposome surfaces is attributed to the close proximity of the reactant. A potential disadvantage of the liposomal system can be, that the overall photooxidation of the photosensitizer at the membrane surface becomes rate limiting, which can be due to a limited access of the water‐soluble reactant to the photosensitizer on the pseudo‐2D‐membrane surface.[^10^, ^41^]

In catalysis with liposomes, temperature effects related to the transition temperature of the phospholipid mixture were observed. Very slow reaction rates and catalytic activity were observed at temperature below the gel‐to‐fluid phase transition temperature which is attributed to the higher mobility of the reactants in the membrane and hence increased diffusion rates.[^18^, ^38^] On the other hand, clustering at lower mobility within the membrane can also increase the catalytic performance, as it was shown for water oxidation in liposomes by König *et al*.^[11]^ Furthermore, rigid hydrophobic environment within the membrane can also protect the individual species from side reactions and therefore increase the catalytic performance in lipid membranes in their more rigid gel phase.^[45]^

Vesicles have three distinguished compartments: the inner aqueous compartment, the hydrophobic membrane, and the aqueous bulk. Natural cell organelles, such as chloroplasts or mitochondria, take advantage of this compartmentalization for all sorts of biological functions, including cellular energy conversion. In the thylakoid membrane, upon photoinduced charge transfer across the membrane and a transmembrane pH gradient is established generating a proton‐motif‐force which provides energy for the ATP synthesis.

**Figure 6:**
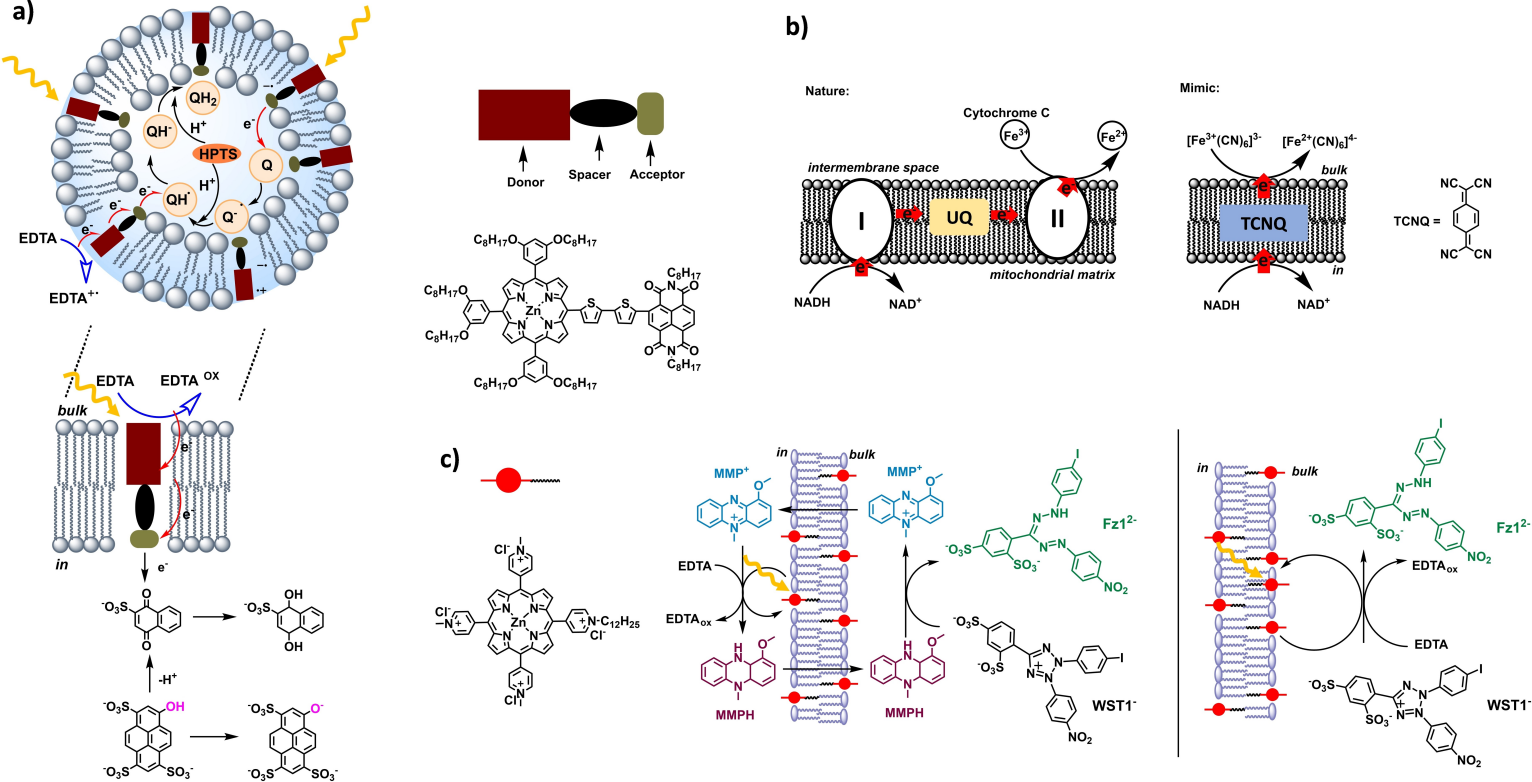
a) Charge separation, transmembrane electron transfer and a proton gradient were achieved with zinc‐porphyrin‐NDI dyads with suitable spacer length matching the length of lipid bilayer membrane. Reproduced with permission from ref. [42]; Copyright: 2015, Elsevier. b) TCNQ acting as electron relay across lipid bilayer membrane replacing transmembrane electron transfer proteins I and II as well as membrane soluble ubiquinone (UQ). Reproduced with permission from ref. [43]; Copyright: 2018, American Chemical Society. c) An amphiphilic, membrane embedded zinc porphyrin is photoreduced by the sacrificial electron donor EDTA in the inner aqueous compartment of liposomes. The membrane soluble electron relay, MMPH, catalytically transports the electrons across the membrane to reduce the WST1^−^ dye in the bulk to generate the strongly colored Fz1^2−^. In another scenario, the photoreduction took place at the membrane‐bulk interface in absence of the transmembrane electron relay. Reproduced from ref. [44]; published 2015 by Royal Society of Chemistry, Creative Commons Attribution 3.0 Unported Licence.

Transmembrane electron transfer and coupled pH‐gradients have been realized in artificial liposomes with molecular systems by various research groups.[^1^, ^6^] In the following we want to highlight some relatively recent examples which are depicted in Figure 6. Bhosale *et al*. have incorporated molecular dyads into the lipid bilayer of vesicles as schematized in Figure 6a.^[42]^ A dyad typically consists of donor‐spacer‐acceptor‐moieties. The authors have used naphthalene diimide as an acceptor and a zinc porphyrin as a donor. Upon usage of a dithiophene spacer, the length of the molecule spanned 3.5 nm and matched the thickness of an egg yolk phosphatidylcholine‐bilayer. Such vesicles were loaded with in the inner compartment with the water‐soluble sulfonato naphthoquinone as electron acceptor, and 8‐hydroxypyrene‐1,3,6‐trisulfonate (HPTS) as spectroscopic pH sensor and proton donor. Upon photoexcitation in presence of the sacrificial reductant EDTA in the bulk, the pH in the inner aqueous compartment increased, because of the following process: Photoexcitation of the dyad was followed by (photo‐)reduction of the dyad by EDTA. The reduced dyad transferred the electrons intramolecularly across the membrane, and the quinone was reduced. The reduced quinone in the inner compartment took up the surrounding protons and basifies the inner compartment. It was furthermore shown that this transmembrane electron transfer only took place if the length of the dyad matched the bilayer thickness.^[42]^

Electron transfer across the lipid bilayer membrane can be performed with membrane‐soluble electron relays such as hydrophobic quinones, 1‐methoxy‐*N*‐methylphenazium (MMP^+^) and tetracyanoquinodimethane (TCNQ).[^43^, ^44^] Sundmacher *et al*. mimicked mitochondrial transmembrane electron transport with liposomes containing reduced nicotinamide adenine dinucleotide (NADH) in their inner aqueous compartment. The encapsulated NADH was then oxidized by the membrane soluble TCNQ and in turn reduced ferricyanide located outside of liposomes.^[44]^ In this study, the simple molecule TCNQ could be used to replace the natural transmembrane enzymes NADH‐ubiquinone oxidoreductase (I) and quinol‐cytochrome‐c oxidoreductase (II) as well as Fe ions in the heme group of cytochrome C (see Figure 6b).

In another system, *light‐induced* transmembrane electron transfer was achieved using DPPC phospholipid bilayer vesicles, functionalized with amphiphilic zinc porphyrins. The sacrificial hydrophilic electron donor EDTA was encapsulated within these vesicles, and upon irradiation with visible light the photoreduction of the porphyrin took place. Electron transfer across the membrane only took place when the electron relay 1‐methoxy‐*N*‐methylphenazium (MMP^+^) was added as methyl sulfate salt: reduction of MMP^+^ yielded the neutrally charged MMPH which is hydrophobic enough to cross the hydrophobic membrane. The water soluble 2‐(4‐iodophenyl)‐3‐(4‐nitrophenyl)‐5‐(2,4‐disulfophenyl)‐2*H*‐tetrazolium anion (WST1^−^) was added to the bulk and was reduced to the strongly colored formazan dye Fz1^2−^. Interestingly, the quantum yield for Fz1^2−^ generation was 2.7 times higher when the direct photoreduction was physically separated from the Fz1^2−^ generation by the vesicle membrane (Figure 6c). The authors attribute this separation of photosensitizer and photoproduct through the membrane leads to prevents recombination reactions in the photoexcited state.^[43]^

## 4 Light‐Energy Conversion with Polymersomes

In order to produce robust material at a large scale for large scale solar fuels production, a more robust material is needed. The discussed lipid vesicle membranes are good and excellent material for biomimetic construction of processes, but their dynamic and relatively labile molecular and material properties might be disadvantageous for large scale solar fuel production. Polymer based vesicles (so called polymersomes) have proven to be very versatile in properties[^46^, ^47^, ^48^, ^49^, ^50^, ^51^, ^52^, ^53^, ^54^, ^55^, ^56^] as well as opportunities for functionalization with proteins such as transmembrane proteins.^[57]^

Polymersome membranes are generally constructed by self‐assembly of individual AB or ABC linear, branched or dendritic copolymers (examples for linear copolymers see Figure 2). Oftentimes, A describes the hydrophilic part, B the hydrophobic part, and C is an optional hydrophilic part. In general, a molecular weight ration of A/B of 1/3 to 1/2 enables for bilayer formation in water, whereas an A/B ration of≤1/3 favors the formation of micelles. This behavior is similar to lipids and based on the packing parameters that govern the geometry of their packing behavior.^[58]^

While lipid‐based vesicles are very well studies for photochemical and artificial photosynthetic reactions, polymersomes are so far much less investigated. It might be the availability of the material, or the specific properties: The rigidity and mechanical robustness of polymer type materials might affect the reaction rates at the membrane surface, as well as in transmembrane processes such as permeation or transmembrane mass transport.

In the following, several studies on light induced/light driven processes will be presented, including selected studies which propose avenues to overcome permeability challenges for light induced processes to in these artificial vesicles.

**Figure 7:**
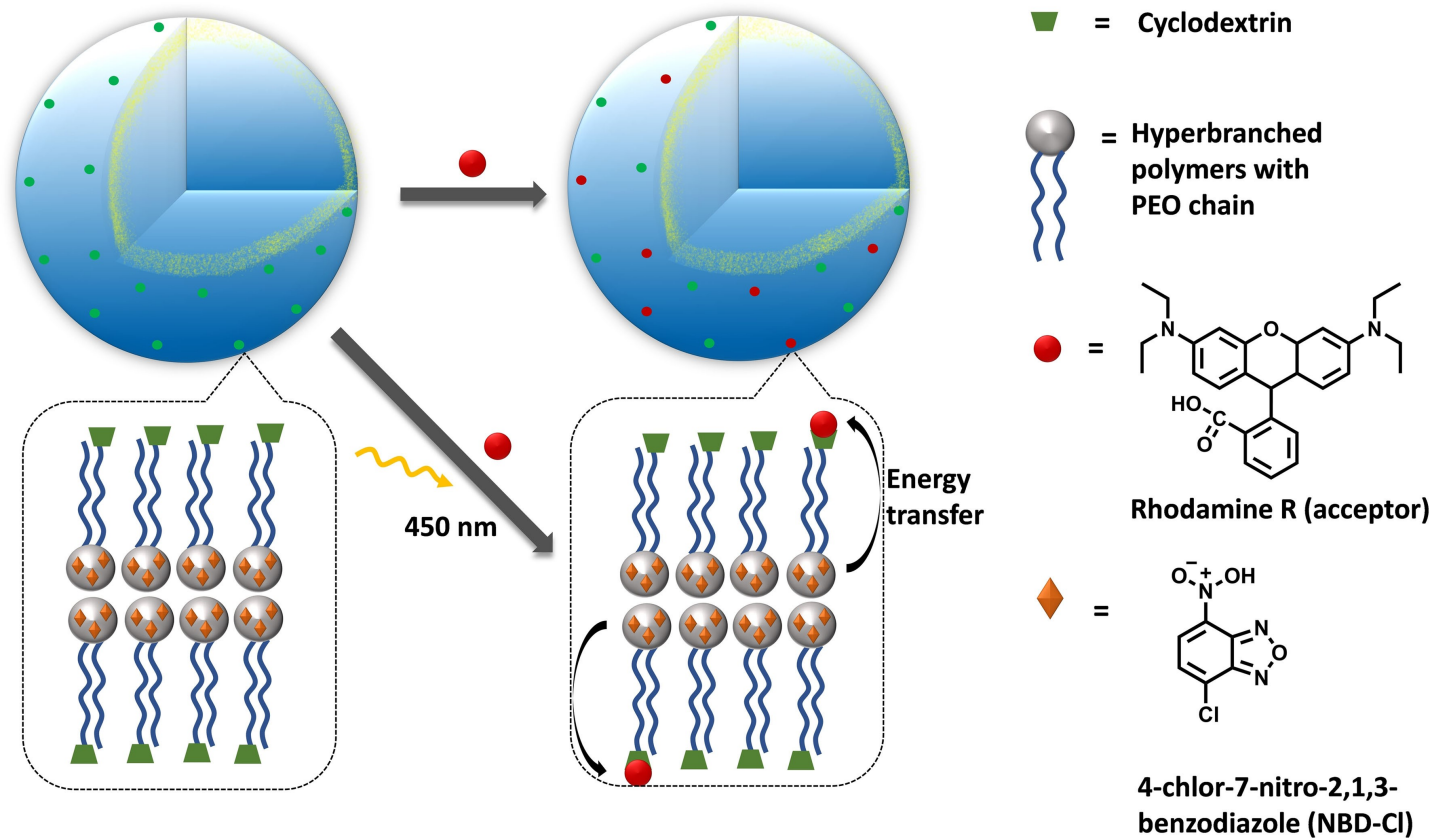
Energy transfer mediated by hyperbranched polymers with NBD‐Cl as energy donor encapsulated inside hyperbranched cores and rhodamine B as energy acceptors incorporated on the surface of vesicle with cyclodextrin as host (PEO=polyethyleneoxide) Reproduced with permission from ref. [59]; Copyright: 2017, John Wiley and Sons.

Zheng, Zhou *et al*. have constructed a light‐harvesting polymeric vesicles with spatially separated donors and acceptors, based on hyperbranched polymers (branched polymersomes, Figure 7).^[59]^ With their system the authors mimicked the natural photosynthetic light harvesting systems, by encapsulating the energy donors 4‐chlor‐7‐nitro‐2,1,3‐benzodiazole (NBD‐Cl) in the hyperbranched cores of the vesicles and anchoring the hydrophilic rhodamine R energy acceptors to the surface of the vesicles via host guest interactions with cyclodextrins. Due to the encapsulation and host‐guest interactions of energy donor and acceptor detrimental self‐quenching is avoided, and the energy transfer efficiency is 80 %.^[59]^

Thordarsen *et al*. have self‐assembled the transmembrane electron transfer protein cytochrome c oxidase of the natural mitochondrial electron transport chain within the hydrophobic part of the polymersome membrane of polystyrene‐polyacrylic acid block copolymer bilayers (see Figure 2).^[61]^ In addition to this natural protein, a semi‐artificial protein complex was associated via self‐assembly: The natural cytochrome c was covalently functionalized at CYS102 with a photoredox active ruthenium terpyridine complex, which was positively charged and situated in the negatively charged, hydrophilic polyacrylic acid layer of the copolymer bilayers. Light irradiation in presence of sacrificial electron donating EDTA enabled for photoreduction of the transmembrane protein, followed by oxygen reduction in the inner compartment of the polymersome and proton translocation from inside to outside of the vesicle, as it is the function of cytochrome oxidase. As a consequence, a basification of the inner compartment was observed via a fluorescent pH marker.^[60]^ This pH gradient is highly biomimetic and indicates the storage of light energy in form of an electrochemical transmembrane potential. It might be used for the driving of ATP synthase.

As described earlier, polymersomes can be constructed of hydrophobic‐hydrophilic block‐copolymers. Typically, the A‐part composes of the hydrophilic part while the B‐part of the co‐polymers consists of the hydrophobic tail. An additional hydrophobic or hydrophilic C‐block‐copolymer might be added as well. To address the issue of mass transport from the inside to the outside of the polymersome, several approaches have been proposed. They include integration of transmembrane proteins,[^61^, ^62^] and reaction‐induced triggering of the permeability properties of the membrane.[^46^, ^47^] The latter is especially interesting in the context of artificial photosynthesis, as the reactions can directly be triggered by light irradiation. Liu *et al*. have applied this context to achieve a transition from typical polymersomes to so called polyion complex vesicles (PICsomes).^[63]^ They incorporated light‐cleavable ester groups, as well as tertiary amine functions into the B and C co‐block polymers. Upon ester cleavage, the carboxylic acid function can be deprotonated by the neighboring tertiary amine an generate ion pairs. In synergy with other hydrogen bonding interactions within the copolymer framework, the ion pair formed a stable membrane that was permeable to water soluble substances. Furthermore, the authors demonstrated that by selectively choosing the composition of the B and C co‐block polymers, the size selectivity could be achieved, which demonstrated, that the challenge of mass transport can be solved with polymeric vesicles.^[63]^

In summary, we have presented several recent studies on light induced electron and energy transfer within liposomal and polymer membranes that will be highly relevant for the construction of artificial photosynthetic membrane systems. We believe that the aspect of compartmentalization of reactions and self‐assembly of reactants will become more and more important for chemistry in general, and light energy conversion in particular. Using vesicles based on biomimetic lipids or artificial polymers will enable various strategies in this regard.

## Conflict of interest

The authors declare no conflict of interest.

## Biographical Information

*Novitasari Sinambela studied chemistry at the Bandung Institute of Technology, Indonesia. She received her MSc degree in 2019. She is currently a PhD candidate in the laboratory of Andrea Pannwitz at Ulm University. Her work focusses on liposomes for artificial photosynthetic purposes, such as photocatalytic water splitting and transmembrane electron transfer*.

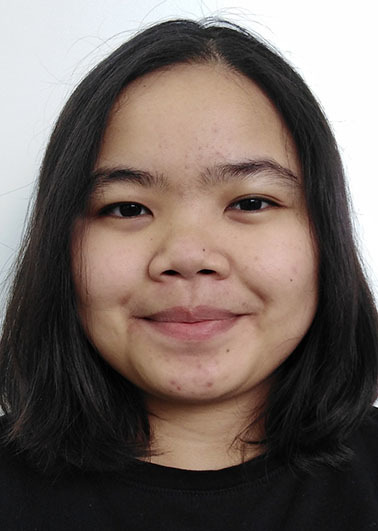

## Biographical Information

*Julian Bösking studied chemistry at Ulm University, Germany. After receiving his MSc degree in 2020 he started his PhD at Ulm University. His work focusses on energy transfer in vesicles*.

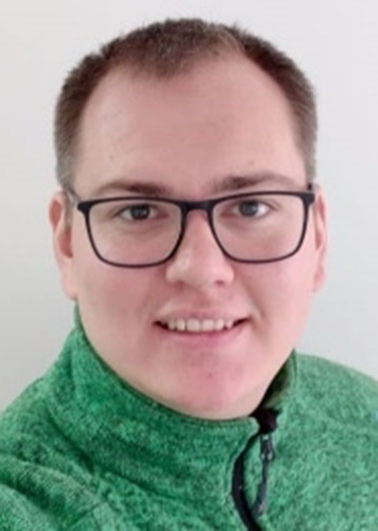

## Biographical Information

*Amir Abbas studied chemistry at the University of Sassari, Italy. He received his MSc degree in 2020 and is currently a PhD candidate in Andrea Pannwitz’ research group at Ulm University on the functionalization of liposomes and polymersome vesicles*.

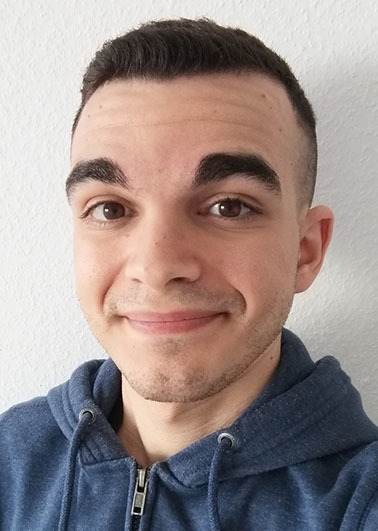

## Biographical Information

*Andrea Pannwitz obtained her PhD in 2017 at the University of Basel in Switzerland. After her Postdoc at Leiden University, The Netherlands, she started her independent career as junior professor for Inorganic Chemistry and Energy Conversion at Ulm University in Germany, with a major interest in photochemical conversions at membrane interfaces*.